# Bioinformatic Analysis of the Protective Effects of Dexmedetomidine and Thrombopoietin Against Hypoxia/Reoxygenation‐Induced Injury in AC16 Cells

**DOI:** 10.1111/cbdd.70105

**Published:** 2025-04-19

**Authors:** Cuiyan Xing, Mingyi Wu, Xiaoyang Zhou, Benhang Gong

**Affiliations:** ^1^ Department of Anesthesiology Shandong Provincial Hospital Affiliated to Shandong First Medical University Jinan Shandong China; ^2^ Department of Anesthesiology, School and Hospital of Stomatology Cheeloo College of Medicine, Shandong University Jinan China

**Keywords:** cardiomyocytes, dexmedetomidine, gene expression regulation, hypoxia/reoxygenation injury, thrombopoietin

## Abstract

This study aimed to investigate the protective mechanisms of dexmedetomidine (Dex) and thrombopoietin (TPO) against hypoxia/reoxygenation (H/R)‐induced myocardial injury. Human cardiomyocyte AC16 cells were subjected to hypoxic conditions and treated with Dex and TPO. Cellular responses, including proliferation, apoptosis, and autophagy, were assessed. RNA sequencing and bioinformatic analyses were conducted to identify differentially expressed genes, followed by functional pathway enrichment analysis. The results demonstrated that Dex and TPO significantly promoted cell proliferation, reduced apoptosis and autophagy, and inhibited caspase‐3 activity and light chain 3B (LC3B) expression. Pathway enrichment analysis revealed the involvement of mitogen‐activated protein kinase (MAPK), transforming growth factor beta (TGF‐β), and tumor necrosis factor (TNF) signaling pathways. Although both treatments demonstrated overlapping effects, they also exhibited distinct gene regulation mechanisms. These findings suggested that Dex and TPO could mitigate H/R‐induced myocardial injury through complex gene regulatory mechanisms, highlighting their potential as therapeutic strategies for myocardial ischemia–reperfusion injury (MIRI).

## Graphical abstract

This diagram illustrates the protective effects of dexmedetomidine (Dex) and thrombopoietin (TPO) against hypoxia/reoxygenation (H/R)‐induced injury in AC16 cells. Both treatments activate signaling pathways, including stem cell pluripotency, TNF, TGF‐β, and MAPK, promoting cell proliferation and inhibiting apoptosis. Dex and TPO suppress caspase‐3 activity and autophagy, providing protection against cellular damage induced by H/R injury.

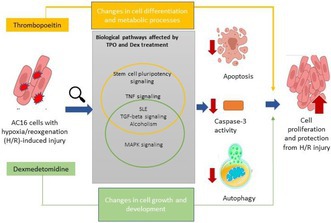

## 1 Introduction

Myocardial ischemia–reperfusion injury (MIRI) is a complex and multifactorial pathophysiological condition that occurs when blood flow is restored to an organ after a period of ischemia, leading to further cellular and tissue damage (Sagris et al. 2024). During ischemia, myocardial cells experience oxygen deprivation and metabolic disturbances, leading to energy metabolism dysfunction and cellular injury (Yellon and Hausenloy 2007). Upon reperfusion, oxygen reenters the ischemic tissue, triggering the production of reactive oxygen species (ROS), which exacerbate oxidative stress and inflammatory responses, leading to the activation of apoptotic and autophagic processes (Wang et al. 2025). These events contribute to further injury, aggravating the initial ischemic damage, particularly for patients undergoing coronary artery bypass grafting, percutaneous coronary intervention, cardiac transplantation, and thrombolysis therapy (Huang et al. 2018). Despite the advancements in these surgical interventions, effective pharmacological therapies to mitigate MIRI and improve patient outcomes remain limited, necessitating further investigation into novel therapeutic strategies (Li et al. 2024).

Dexmedetomidine (Dex), a selective α2‐adrenergic receptor agonist, has been widely used in clinical practice as an anesthetic and sedative (Lee 2019). In recent years, there has been growing evidence suggesting that Dex exerts significant cardioprotective effects, particularly in the context of ischemia–reperfusion injury (Gao et al. 2017; Ibacache et al. 2012). Preconditioning with Dex has been shown to reduce myocardial injury by attenuating oxidative stress, modulating inflammatory pathways, and inhibiting apoptosis (Ji et al. 2013; Yi et al. 2018). Dex's protective mechanism involves the activation of pro‐survival signaling pathways, such as the mitogen‐activated protein kinase (MAPK) and phosphatidylinositol‐3‐kinase/protein kinase B (PI3K/Akt) pathways, although the precise molecular mechanisms remain unclear (Jiang et al. 2024). Understanding how Dex works at the molecular level could provide insights into its therapeutic potential for MIRI.

Thrombopoietin (TPO), primarily known for its role in platelet production, has also emerged as a critical cytokine involved in tissue repair and protection (Baker et al. 2015). Recent studies have demonstrated that TPO offers direct protection against ischemia–reperfusion injury in various organs, including the heart and brain (Baker et al. 2008; Zhou et al. 2011). TPO activates several intracellular signaling pathways, including JAK/STAT, MAPK, and PI3K/Akt, which are involved in regulating apoptosis, inflammation, and cell proliferation (Bhat et al. 2018). TPO has also been shown to repair neuronal endothelial cell formation (Varghese et al. 2017). Despite these promising findings, the specific protective mechanisms of TPO in cardiomyocytes, especially under myocardial hypoxia/reoxygenation (H/R) conditions, remain poorly understood. Therefore, investigating the potential therapeutic benefits of TPO for MIRI is of significant interest.

The present study aimed to evaluate the protective effects of Dex and TPO against H/R‐induced myocardial injury in AC16 cardiomyocyte cells, a widely used in vitro model of MIRI. By utilizing RNA‐sequencing (RNA‐seq) and bioinformatic analyses, we examined the differential gene expression profiles and identified key signaling pathways regulated by Dex and TPO. This study seeks to provide deeper insights into the molecular mechanisms through which these agents mitigate H/R‐induced injury, contributing to the development of potential therapeutic strategies for MIRI.

## 2 Methods

### 2.1 Cell Culture and Trial Grouping

The human cardiomyocyte cell line AC16 was purchased from Shanghai Cell Bank at the Chinese Academy of Science (Shanghai, China). Cells were cultured in Dulbecco's modified Eagle's medium (DMEM) supplemented with 1% (v/v) penicillin–streptomycin (Beyotime Biotech., Shanghai, China) and 10% fetal bovine serum (FBS, Gibco BRL, NY, USA) at a density of 1 × 10^5^ cells/well in 96‐well plates. The cultures were maintained at 37°C in a humidified incubator (Mode: MCO‐15 AC‐SC, Sanyo, Tokyo, Japan) with 5% CO_2_ for 24 h. Following this incubation, cells were treated with Dex (Jiangsu Hengrui Medicine Co. Ltd., Jiangsu, China) dissolved in DMSO, or TPO (PeproTech, NJ, USA) for 30 min.

The cells were divided into three groups: Control (CNTL), Dex, and TPO. Within the Dex group, six subgroups were treated with increasing concentrations of Dex (0.1, 0.3, 1.0, 3.0, 10.0, and 30 nM). The TPO group was similarly divided into six subgroups, with concentrations of 0.1, 0.3, 1.0, 3.0, 10.0, and 30 ng/mL of TPO. Each experimental condition was performed in triplicate.

For H/R injury, AC16 cells were cultured in glucose‐free DMEM supplemented with Dex or TPO for 30 min. Subsequently, the cells were subjected to hypoxic conditions (95% nitrogen and 5% CO_2_) at 37°C for 5 h, followed by reoxygenation under 5% CO_2_ for 16 h.

### 2.2 Cell Proliferation Assay

The proliferative rates of AC16 cells were evaluated using the Cell Counting Kit‐8 (CCK‐8, Cat# 96992, Sigma‐Aldrich, USA) according to the manufacturer's instructions. Briefly, AC16 cells were cultured in 96‐well plates (Corning, NY, USA) at a density of 6000 cells/well and treated with different concentrations of Dex or TPO. After exposure to H/R conditions, cells were cultured for 0, 12, and 24 h. CCK‐8 solution was added to each well, and incubation continued for 4 h. The optical density was measured at 450 nm with a microplate reader (Thermo Fisher Scientific, MA, USA). The cell proliferation rate (%) was calculated as the percentage of inhibition relative to the CNTL group.

### 2.3 Apoptosis Assay

Apoptosis in AC16 cells under H/R conditions was evaluated by flow cytometry, Hoechst 33342 staining, and caspase‐3 activity.

For Hoechst 33342 staining, cells were fixed in 4% formaldehyde (Sangon Biotech Co. Ltd., Shanghai, China) at 4°C for 10 min and then incubated with Hoechst 33342 (Cat# C1022, Beyotime Biotech.) at a final concentration of 5 μg/mL in the dark for 10 min. The stained AC16 cells were examined under a laser‐scanning confocal microscope (Nikon A1R, Tokyo, Japan) with excitation at 350 nm and emission at 460 nm.

Apoptosis was quantified using the Annexin V‐PE/7‐AAD Flow Cytometry Kit (Cat#559763, BD Biosciences, NJ, USA) to evaluate the rate of apoptosis following the manufacturer's instructions. Cells were washed three times with D‐Hanks buffer (Beyotime Biotech.) at 4°C for 5 min and adjusted to a density of 1 × 10^6^ cells/mL. Next, 7‐AAD (5 μL) and Annexin V‐PE (1 μL) were added to 100 μL of cell suspension per well, followed by incubation at room temperature for 10 min in the dark. After adding binding buffer (450 μL), cells were analyzed using a FACSCalibur flow cytometer (BD Biosciences). Apoptotic cells were identified based on Annexin V‐PE and 7‐AAD staining.

Caspase‐3 activity was evaluated using the Caspase‐3 Fluorometric Assay Kit (Cat# K105‐25, BioVision, CA, USA). The cells were removed from the plates by digestion with trypsin (Cat# C0201, Beyotime Biotech.) and collected by centrifugation at 600×*g* for 5 min. The cells were lysed on ice for 15 min, and cell debris was pelleted by centrifugation at 20,000×*g* for 15 min at 4°C. The supernatants were collected to evaluate caspase‐3 activity according to the manufacturer's instructions.

### 2.4 Measurement of Light Chain 3B (LC3B) Expression

Autophagy was assessed by quantifying LC3B expression in AC16 cells. Cells were seeded onto cover slides at a density of 1 × 10^5^ cells/slide and cultured for 24 h. Following treatment with Dex or TPO, cells were washed with phosphate‐buffered saline (PBS, Beyotime Biotech.), prefixed with 4% paraformaldehyde (Beyotime Biotech.) for 15 s, and then permeabilized with ice‐cold methanol (Sinopharm Chemical Reagent Co. Ltd., Shanghai, China) at −20°C for 10 min. The prepared slides were washed with PBS and air‐dried at 24°C. The slides were incubated with rabbit anti‐human LC3B (1:1000, Cat# ab168831, Abcam, Cambridge, UK) overnight at 4°C. The slides were continuously washed three times with PBS for 5 min and incubated with fluorescein isothiocyanate‐conjugated goat anti‐rabbit antibody (1:800, Cat# A0562, Beyotime Biotech) at 37°C for 30 min. The stained slides were observed under a fluorescence microscope (Mode: H600L, Nikon, Tokyo, Japan). The number of LC3B‐positive dots was evaluated and quantified using the FV10‐ASW 3.0 Image software (Olympus, Tokyo, Japan).

### 2.5 RNA Isolation

AC16 cells were initially cultured in T25 flasks with Ready‐to‐Use Myocyte Growth Medium (Cat# C‐22060, PromoCell GmbH, Heidelberg, Germany) supplemented with 20% FBS (PromoCell GmbH) for 24 h. The cell medium was replaced with DMEM containing 17% medium 199 (Cat# 11150059, Gibco BRL), 10% horse serum (Gibco BRL), 5% FBS (Gibco BRL), and 0.5% penicillin–streptomycin (Beyotime Biotech.). After pretreatment with Dex or TPO for 30 min, AC16 cells were subjected to hypoxic conditions at 37°C for 5 h and then reoxygenated in 5% CO_2_ for 16 h. Total RNA was extracted using the TRIzol reagent (Cat#R0016, Beyotime Biotech.) according to the manufacturer's instructions. Total RNA was quantified using a spectrophotometer (Mode: UV754N, Shanghai Aucy Sci. Ins. Ltd., Shanghai, China). RNA integrity and purity were assessed using an RNA 6000 Nano LabChip Kit (Cat# 5067‐1511) and Bioanalyzer (Mode: Agilent 2100, Agilent Technologies, CA, USA). The RNA integrity number was ≥ 9 and the 260/280 ratio was ≥ 2.0 for all samples.

### 2.6 RNA‐Sequencing

RNA‐seq was performed as previously described (Florea et al. 2013) with slight modifications. The first and second strand cDNA were synthesized using 6‐base random primers, dNTPs, RNase H, and DNA polymerase I (Thermo Fisher Scientific). cDNA was purified and washed using a QIAQuick PCR Purification Kit (Cat# 28104, Qiagen, Hilden, Germany) before undergoing end‐repair, 3′ adenylation, and ligation of a sequencing adapter using a TruSeq RNA Sample Preparation Kit (Cat# RS‐122‐2503, Illumina, San Diego, CA, USA). Purified cDNA was recovered by agarose gel electrophoresis, and PCR was performed to obtain the whole‐transcriptome libraries. Finally, the samples were sequenced using the PE150 protocol. Sequencing depth was set to 30–50 million paired‐end reads per sample, with a minimum read length of 75–100 base pairs required to ensure sufficient coverage and accurate identification of alternative splicing events, as well as reliable detection of gene expression levels.

### 2.7 Computational Analysis and Gene Functional Annotation

Raw sequence data were processed using FastQC (Version 0.11.9) to assess the quality of reads, followed by trimming of low‐quality sequences and removal of adapter sequences using Trim Galore (Version 0.6.5) with the following parameters: minimum read length of 50 bp and quality score threshold of 30. High‐quality reads were then aligned to the human reference genome (GRCh38) using HISAT2 (Version 2.1.0) with default settings. Differential expression analysis was performed on the aligned data using the EdgeR (version 3.28.1) package, which uses a Poisson‐based model to assess RNA‐seq data variance.

Gene functional analysis annotation was performed using the Database for the Annotation, Visualization and Integrated Discovery (DAVID, Version 6.7) for gene ontology (GO) and Kyoto Encyclopedia of Genes and Genomes (KEGG) enrichment analysis. GO terms were considered enriched when their *p*‐value was < 0.05. For KEGG analysis, the KEGG pathways with a false discovery rate (FDR) < 0.05 were considered significantly enriched. All parameters and methods used in this analysis were previously described (Herrer et al. 2014; Kanehisa et al. 2016; Paquette and Tokuyasu 2010).

### 2.8 Alternative Splicing Analysis

Alternative splicing is a process that transforms mRNA precursors into multiple mRNA isoforms by differential splicing or different splice sites (Florea et al. 2013). The OLego mapping method and Quantas pipelines were applied to the RNA‐seq data to identify potential alternative splicing events according to a previously published workflow (Wu et al. 2013).

### 2.9 Gene Variation Analysis

Gene variants, including single‐nucleotide polymorphisms (SNPs) and insertions/deletions (InDels), were identified using Samtools by comparing the aligned RNA‐seq data to the reference genome (Li et al. 2009). The distribution and frequency of these variants were analyzed, with results compared to previously published data on gene variation in response to treatments (Boland et al. 2013).

### 2.10 Statistical Analysis

Statistical analyses were performed using SPSS software 20.0 (SPSS Inc., Chicago, IL, USA). Normality was assessed using the Shapiro–Wilk test, and homogeneity of variances was tested with Levene's test. Data were represented as mean ± standard deviation (SD). For comparisons between more than two groups, one‐way analysis of variance (ANOVA) was used, followed by Tukey's post hoc test to identify pairwise differences. For two‐group comparisons, Student's *t‐*test was applied. Each experiment was conducted with at least three independent biological replicates. A *p* < 0.05 was considered statistically significant.

## 3 Results

### 3.1 Dex and TPO Promote Proliferation in H/R‐Treated AC16 Cells

**FIGURE 1:**
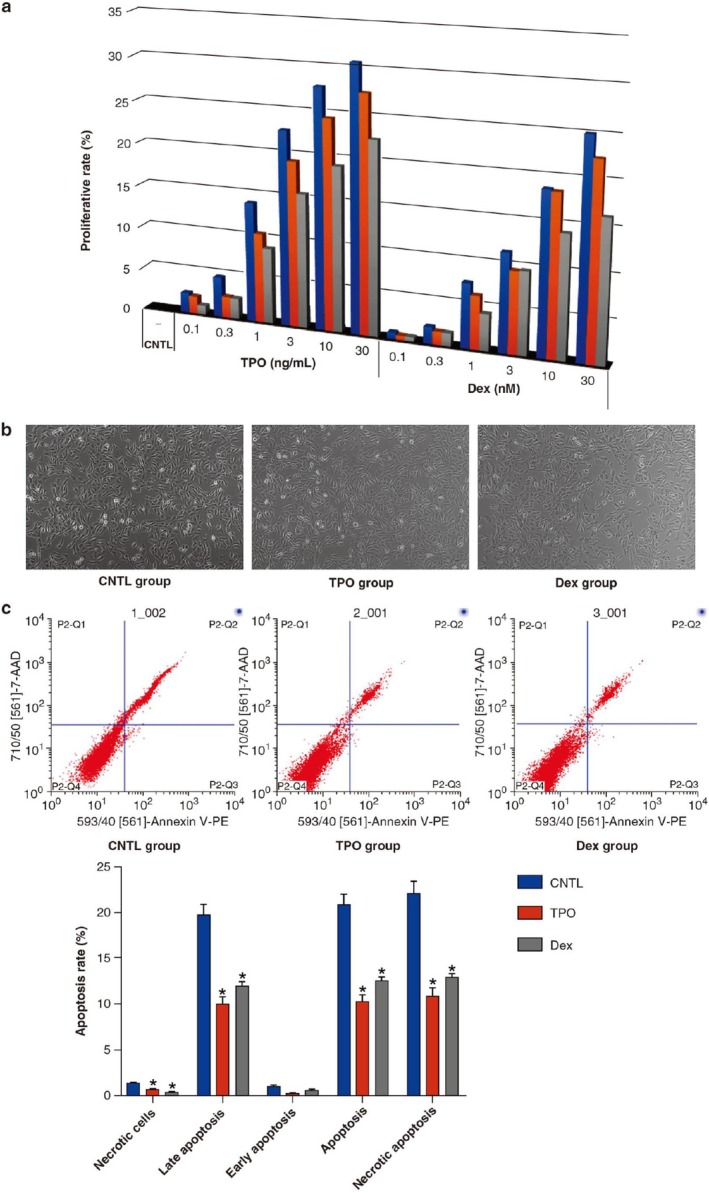
Dex and TPO promote proliferation and inhibit apoptosis of AC16 cells. (a) Effect of Dex and TPO on AC16 cell proliferation. (b, c) Effects of Dex and TPO on AC16 cell apoptosis. *p < 0.05 for comparisons with the CNTL group. CNTL, control; Dex, dexmedetomidine; TPO, thrombopoietin.

AC16 cells were successfully cultured, and the effects of Dex or TPO were evaluated following H/R injury by using the CCK‐8 assay. Compared with the CNTL group, the proliferation rate of AC16 cells treated with TPO increased significantly in a dose‐dependent manner (Figure 1a, *p* < 0.05). At the indicated concentration of TPO, the rate of proliferation decreased with increasing culture time ranging from 0 to 24 h (Figure 1a, *p* < 0.05). The proliferation rate of AC16 cells treated with Dex similarly increased in a dose‐dependent manner (Figure 1a, *p* < 0.05) and decreased with increasing culture time (Figure 1a, *p* < 0.05). The proliferation rates of H/R‐treated AC16 cells in the TPO group were significantly higher than those in the Dex group (Figure 1a, *p* < 0.05). This suggested that TPO and Dex could protect AC16 cells against H/R injury. Based on the results of the CCK‐8 assays, 10 ng/mL TPO and 10 nM Dex were used in subsequent experiments.

### 3.2 Dex and TPO Inhibit Apoptosis and Caspase‐3 Activity in H/R‐Treated AC16 Cells

Dex and TPO significantly improved the morphology of AC16 cells treated with H/R by reducing cellular shrinkage and restoring normal cell shape (Figure 1b). The percentage of necrotic cells, early apoptotic cells, and late apoptotic cells was evaluated by flow cytometry. The percentages of necrotic cells, early apoptotic cells, late apoptotic cells, and total apoptotic cells in the Dex and TPO groups was significantly decreased as compared to those in the CNTL group (Figure 1c, all *p* < 0.05). The proportion of necrotic and apoptotic cells in the Dex group was significantly higher than that in the TPO group (Figure 1c, *p* < 0.05).

**FIGURE 2:**
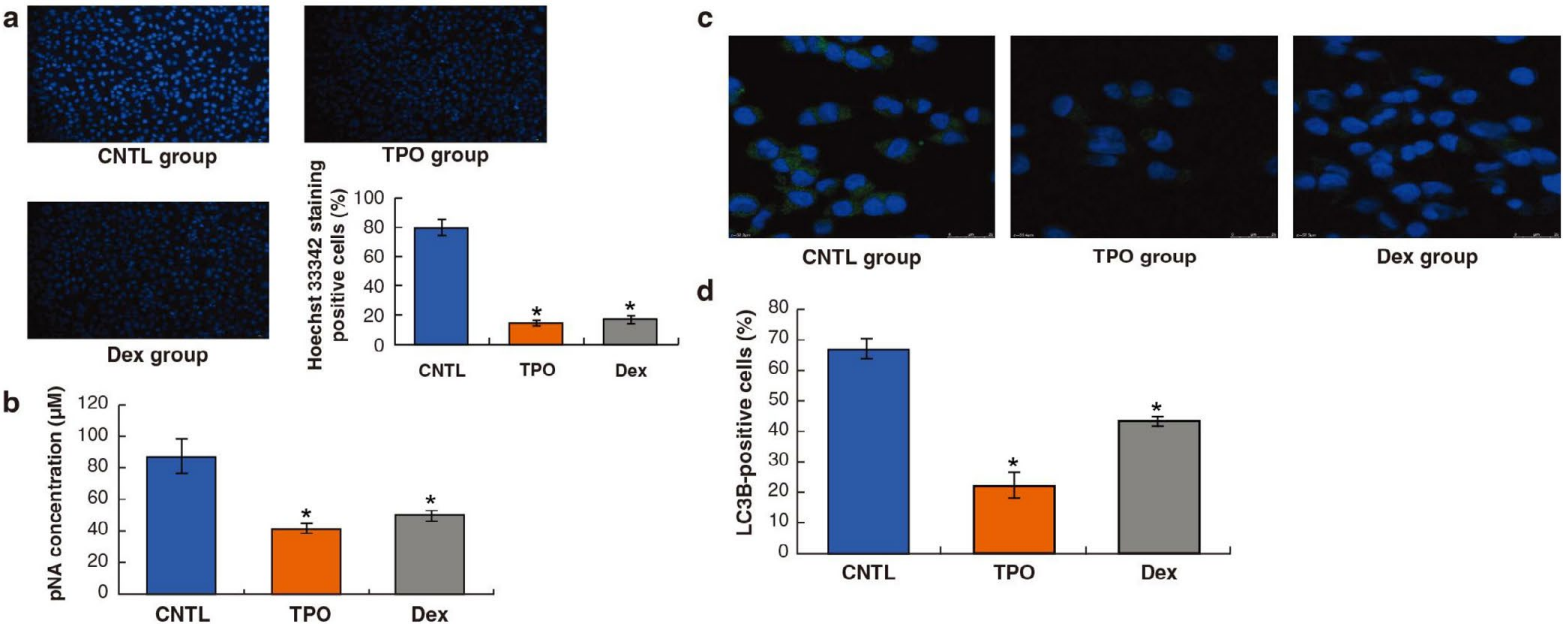
Dex and TPO reduce caspase‐3 activities and autophagy in AC16 cells with H/R injury. (a, b) Effect of Dex and TPO on caspase‐3 activity. (c, d) Effects of Dex and TPO on autophagy. *p < 0.05 for comparisons with the CNTL group. CNTL, control; Dex, dexmedetomidine; TPO, thrombopoietin.

Hoechst 33342 staining in the Dex and TPO group decreased significantly compared to that in the CNTL group (Figure 2a, *p* < 0.05). The caspase‐3 activity indicated by the concentration of p‐nitroaniline in the Dex and TPO groups was also significantly reduced as compared to that in the CNTL group (Figure 2b, *p* < 0.05). These results indicated that TPO and Dex could ameliorate cell death caused by H/R injury.

### 3.3 Dex and TPO Reduce Autophagy in H/R‐Treated AC16 Cells

Autophagy is a type of programmed cell death, the specific biomarker of which is LC3B (Song et al. 2017). Compared with the CNTL group, the numbers of LC3B‐positive cells in the Dex and TPO groups were significantly decreased (Figure 2c,d, *p* < 0.05). This result suggested that Dex and TPO could inhibit autophagy caused by H/R.

### 3.4 Dex and TPO Induce Differential Gene Expression in AC16 Cells

**FIGURE 3:**
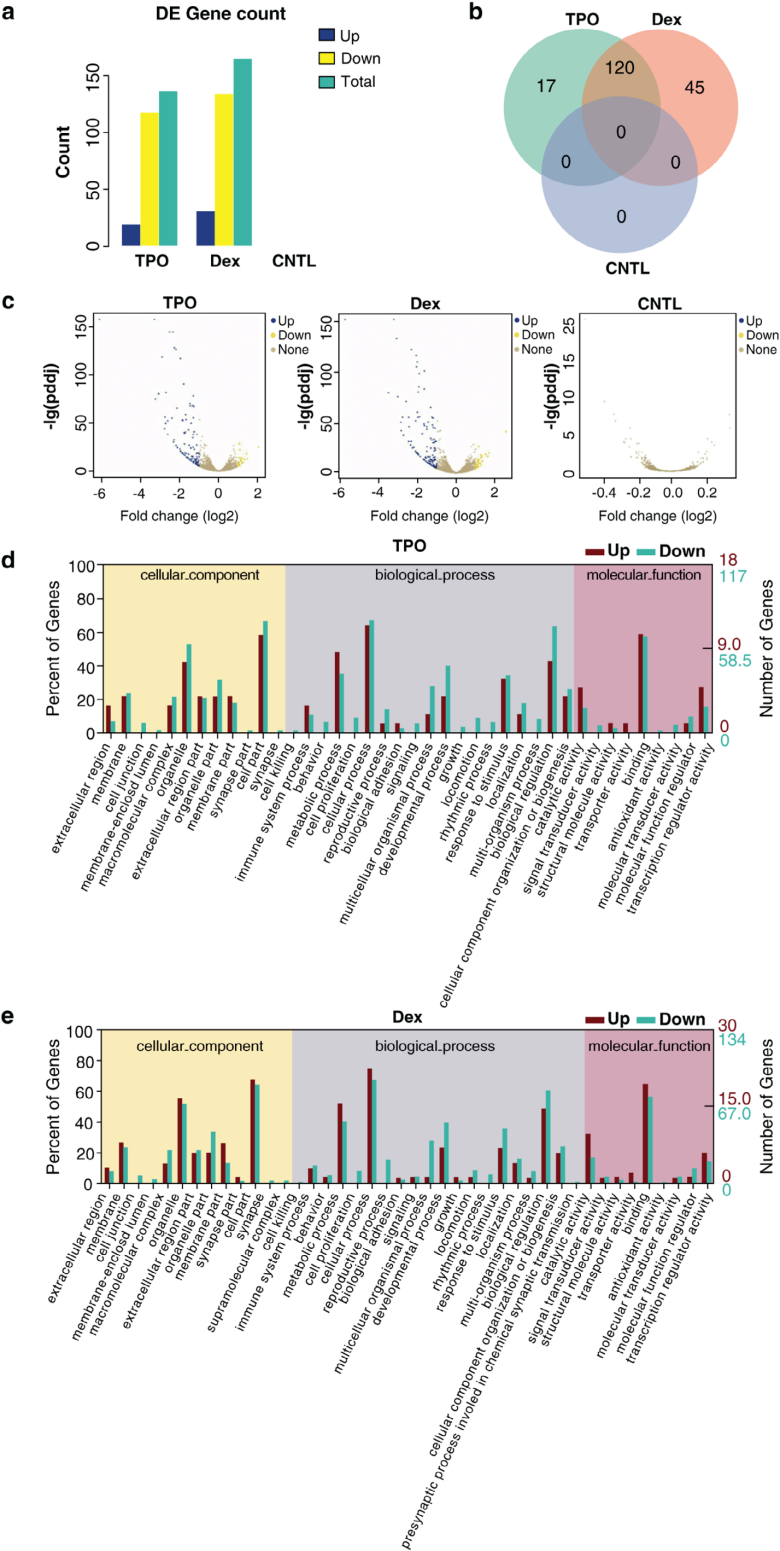
Dex and TPO induce differential expression of genes in AC16 cells with H/R injury. (a) Number of differentially expressed genes in AC16 cells. (b) Overlap of differentially expressed genes in different groups. (c) Volcano plot of differentially expressed genes in different groups. (d)  GO functional analysis of differentially expressed genes in the TPO group. (e) GO functional analysis of differentially expressed genes in the Dex group. CNTL, control; DE, differentially expressed; Dex, dexmedetomidine; TPO, thrombopoietin.

Compared with the CNTL group, 31 upregulated genes and 134 downregulated genes were identified in the Dex group, while the TPO group exhibited 19 upregulated genes and 118 downregulated genes (Figure 3a). Importantly, while there were no significantly differentially expressed genes (DEGs) between the Dex and TPO groups, the analysis revealed that both treatments shared 120 DEGs, indicating some common molecular responses. In addition, there were 17 unique DEGs in the TPO group and 45 unique DEGs in the Dex group (Figure 3b), highlighting the distinct molecular mechanisms influenced by each treatment. The volcano plots and gene Venn diagram (Figure 3c) further illustrated a similar distribution of gene expression levels and statistical significance between the Dex and TPO groups, reinforcing the observation of both shared and unique DEGs.

### 3.5 GO Functional Analysis

The GO functional analysis revealed that there were 12 genes related to cellular component (CC), 19 genes related to biological process (BP), and 9 genes related to molecular function (MF) in the TPO group (Figure 3d). In comparison, the Dex group showed 13 genes for CC, 20 genes for BP, and 9 genes for MF (Figure 3e). Both groups exhibited similar GO terms, gene counts, and gene percentages, indicating that the effects of TPO and Dex on gene regulation share some commonalities. Among the DEGs, the most significantly altered genes were cell part genes in the CC category, cellular process genes in the BP category, and binding genes in the MF category (Figure 3d,e).

### 3.6 GO Enrichment Analysis

GO enrichment analysis identified terms enriched for the differentially regulated proteins unique to the TPO group and to the Dex group. The enriched terms for the TPO group included negative regulation of cellular metabolic process, cell differentiation, and negative regulation of metabolic process. The enriched terms for the Dex group included regulation of multicellular organismal development, developmental process, and negative regulation of multicellular organismal process. This suggests that TPO treatment causes changes in cell differentiation and metabolic processes, whereas Dex treatment causes changes in cell growth and development.

### 3.7 KEGG Pathway Enrichment Analysis

**FIGURE 4:**
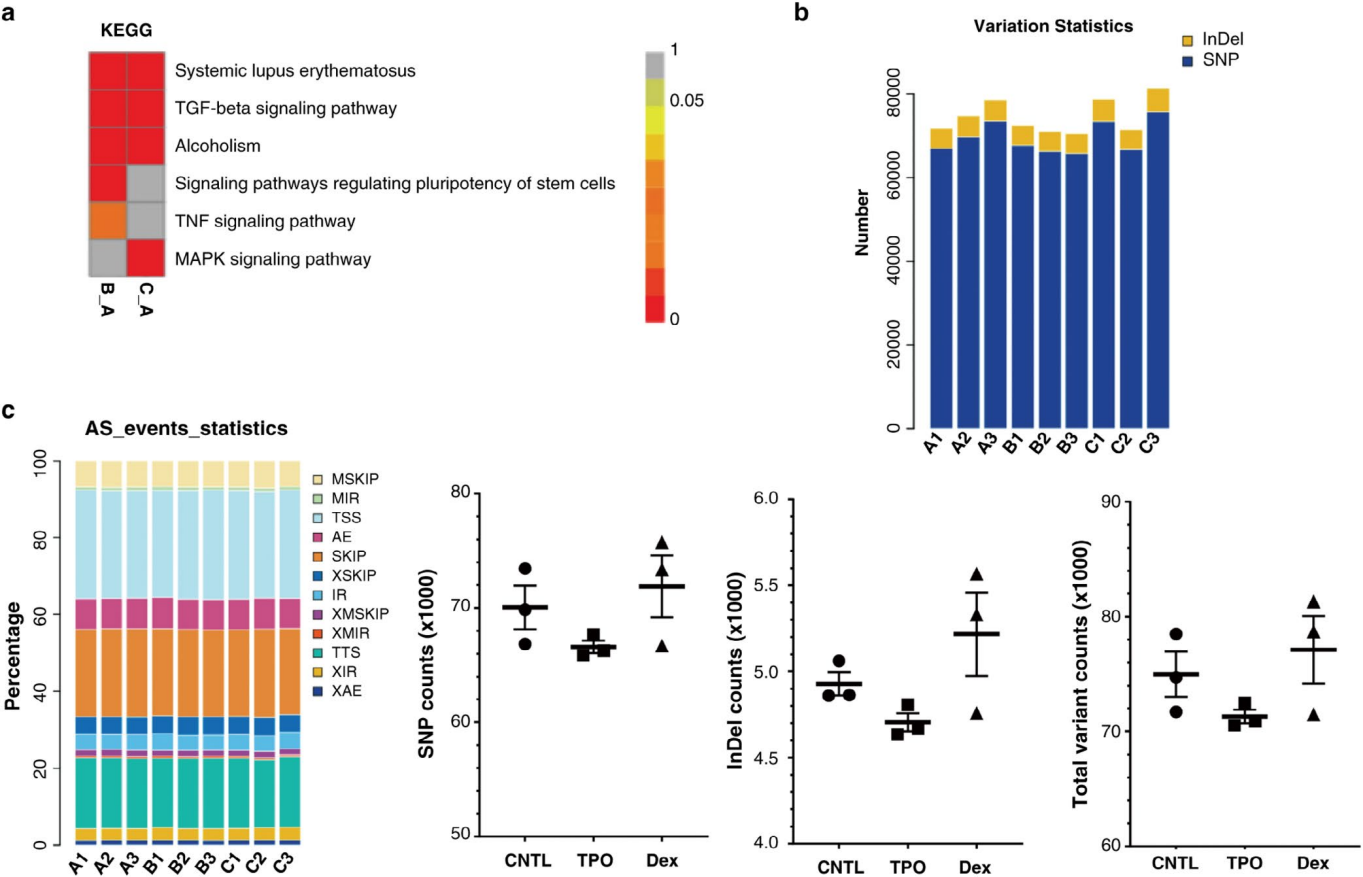
(a) KEGG pathway enrichment analysis in Dex and TPO groups of AC16 cells. (b) Dex and TPO had no effect on splicing events in AC16 cells with H/R injury. (c) Effects of Dex and TPO on the counts of SNP, InDel, and total events. KEGG, Kyoto Encyclopedia of Genes and Genomes; SNPs, single‐nucleotide polymorphisms.

We performed KEGG pathway enrichment analysis to identify biological pathways associated with TPO and Dex treatment. We identified five enriched KEGG pathways based on the unique dysregulated genes in the TPO group, including systemic lupus erythematosus, TGF‐β signaling pathway, alcoholism, and signaling pathways (Figure 4a). Four KEGG pathways showed significant enrichment of DEGs in the Dex group, including MAPK signaling pathway, alcoholism, systemic lupus erythematosus, and TGF‐β signaling pathway (Figure 4a). Systemic lupus erythematosus, TGF‐β signaling pathway, and alcoholism were common KEGG pathways. The signaling pathways regulating pluripotency of stem cells and TNF signaling pathway were unique KEGG pathways in the TPO group. Notably, the Dex group uniquely involved the MAPK signaling pathway (Figure 4a).

### 3.8 Effects of Dex and TPO on Alternative Splicing of AC16 Cells Exposed to H/R

There were no differences in the alternative splicing events and alternative splicing counts between the CNTL, Dex, and TPO groups (Figure 4b, *p* > 0.05). Therefore, Dex and TPO treatment did not result in alternative splicing in AC16 cells.

### 3.9 Effects of Dex and TPO on SNP, InDel, and Total Mutations in AC16 Cells Exposed to H/R

**FIGURE 5:**
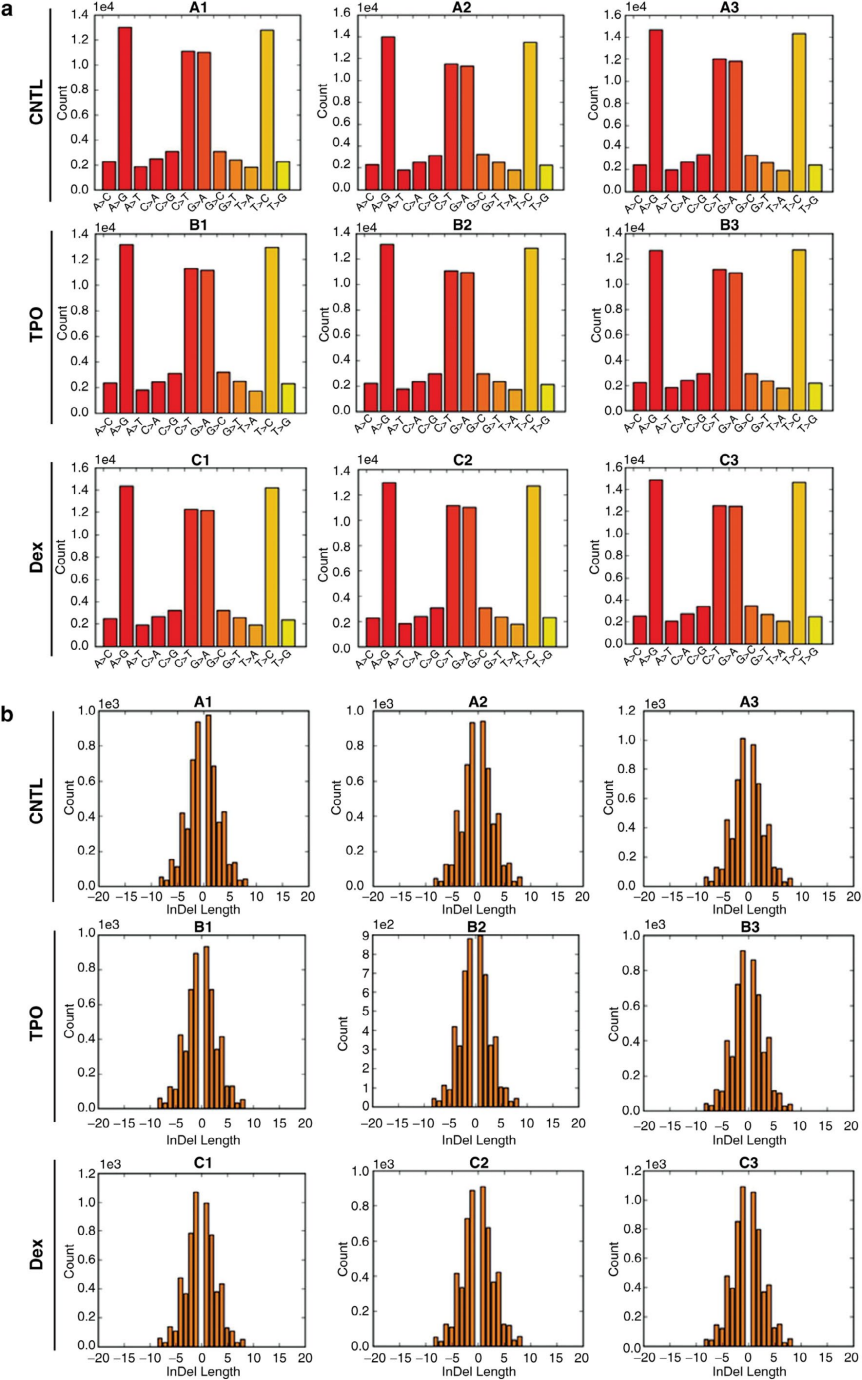
Dex and TPO had no effect on the frequency distribution of SNP mutations (a) or InDel length (b) in AC16 cells with H/R injury. CNTL, control; Dex, dexmedetomidine; TPO, thrombopoietin.

There were no significant differences in the frequency of gene variants, including SNP, InDel, and total mutations, among the CNTL, Dex, and TPO groups (Figure 4c, *p* > 0.05). Neither Dex nor TPO treatment affected the frequency distribution of SNP mutations (Figure 5a, *p* > 0.05). There were no significant differences in the InDel length between the CNTL, Dex, and TPO groups (Figure 5b, *p* > 0.05). Taken together, Dex and TPO did not cause changes in the frequency of gene mutations in AC16 cells exposed to H/R.

## 4 Discussion

In the present study, we demonstrated that Dex and TPO promoted the proliferation of AC16 cells subjected to H/R injury in a concentration‐dependent manner. This protection against H/R‐induced injury is consistent with previous reports showing the cardioprotective effect of Dex (Ammar et al. 2016; Jia et al. 2017; Yang et al. 2017) and TPO (Baker et al. 2015; Bethel et al. 2015). Both treatments significantly suppressed apoptosis and autophagy in AC16 cells exposed to H/R, a result likely due to the inhibition of caspase‐3 activity, a key marker of apoptosis.

The use of the H/R model in AC16 cells is particularly relevant for studying MIRI. MIRI is a complex process that occurs when blood supply to the heart is restored after a period of ischemia. The reintroduction of oxygen leads to oxidative stress, cellular damage, and apoptosis, making it an ideal model for investigating cardioprotective strategies. AC16 cells, as a human‐derived cardiomyocyte cell line, offer a relevant in vitro system for mimicking these pathophysiological conditions (Huang et al. 2023). Our findings provide valuable insight into how Dex and TPO mitigate the damage caused by such injury, suggesting their potential as therapeutic agents for MIRI.

To elucidate the underlying mechanisms of Dex and TPO in H/R injury, we performed a bioinformatics analysis using RNA‐seq. Although there were no DEGs between the Dex and TPO groups, we identified 120 shared DEGs between the two treatments. These common genes, combined with the GO function analysis, suggested that Dex and TPO share similar mechanisms of action at the transcriptional level, particularly in regulating CC, BP, and MF. However, the analysis also revealed unique gene signatures for each treatment, indicating that Dex and TPO have distinct molecular actions. For instance, Dex treatment was enriched in genes related to “multicellular organismal development,” which is consistent with previous studies (Kuter 2013; Lupia et al. 2012). In contrast, TPO uniquely regulated genes involved in platelet function and transcription factor activation, including human platelet glycoprotein 6, forkhead‐related family of mammalian transcription factor 1, and GATA‐family transcription factors by activating demethylation processes (Kanaji et al. 2005; Tanaka et al. 2001), suggesting its role in hematopoiesis and inflammation.

Further functional annotation of the DEGs using KEGG pathway enrichment analysis revealed both shared and unique pathways modulated by Dex and TPO. Both treatments enriched pathways associated with systemic lupus erythematosus, TGF‐β signaling, and alcoholism. Interestingly, while the TPO group uniquely enriched pathways related to the regulation of stem cell pluripotency and TNF signaling, the MAPK signaling pathway was uniquely enriched in the Dex‐treated group. The MAPK pathway is well known for its role in cell survival, inflammation, and apoptosis. Previous studies have indicated that Dex can activate the MAPK pathway to protect cardiomyocytes from ischemia/reperfusion injury (Li et al. 2019). This suggests that the MAPK pathway plays a crucial role in the protective effects of Dex against H/R‐induced injury.

The enrichment of the TGF‐β pathway in both Dex and TPO groups is particularly noteworthy. TGF‐β is a pleiotropic cytokine involved in various cellular processes such as cell differentiation, proliferation, and apoptosis (Liu et al. 2018; Zhang et al. 2014). Its involvement in both treatments points to its central role in mediating the cardioprotective effects of Dex and TPO. Moreover, the unique enrichment of TNF signaling pathways in the TPO‐treated group, which includes key molecules such as nuclear factor κB (NF‐κB) and PI3K/Akt, suggests that TPO may modulate inflammation and cell survival pathways to promote cardioprotection (Abdelwahid et al. 2016; Chen and Goeddel 2002; Foulquier et al. 2018; Jackman et al. 2009; Li et al. 2017; Zhu et al. 2018). These findings provide a deeper understanding of the distinct yet complementary roles of Dex and TPO in mitigating H/R injury.

Additionally, our results indicate that both Dex and TPO do not significantly affect the rate of alternative splicing or gene mutations in AC16 cells undergoing H/R injury, suggesting that their protective effects are not likely mediated by DNA‐level changes. This observation aligns with previous studies that have shown that Dex and TPO primarily act through cellular signaling pathways rather than through genetic mutations or alternative splicing events (Fu et al. 2025; Sun et al. 2024).

In comparison to previous research, our study further elucidates the potential overlap and divergence in the mechanisms of Dex and TPO. While both agents share similar pathways such as TGF‐β and systemic lupus erythematosus, they also exhibit unique molecular actions, particularly in the MAPK pathway for Dex and the stem cell regulation and TNF pathways for TPO. This comparison underscores the potential of these agents in targeting multiple cellular pathways to confer protection against ischemia/reperfusion injury. Further studies are required to validate these findings in other cell lines and animal models of MIRI.

In conclusion, Dex and TPO offer promising strategies for the treatment of MIRI by promoting cell proliferation, inhibiting apoptosis, and reducing autophagy. Bioinformatics analysis indicated that the protective effect of Dex and TPO is mediated through complex, overlapping, and distinct molecular pathways. Further research should explore how these signaling networks interact in vivo and whether Dex and TPO can be translated into clinical therapies for MIRI. Furthermore, the findings of this study highlight the advantages of using Dex in clinical anesthesia, particularly in the context of cardiac surgery, where minimizing myocardial injury is crucial.

## Ethics Statement

The authors have nothing to report.

## Consent

The authors have nothing to report.

## Conflicts of Interest

The authors declare no conflicts of interest.

## Data Availability

The datasets used and analyzed in the current study are available upon reasonable request from the corresponding author.
